# Genome Sequencing of Sub-Arctic Mesomycetozoean Sphaeroforma sirkka Strain B5, Performed with the Oxford Nanopore minION and Illumina HiSeq Systems

**DOI:** 10.1128/MRA.00848-18

**Published:** 2018-10-18

**Authors:** A.-L. Ducluzeau, J. R. Tyson, R. E. Collins, T. P. Snutch, B. T. Hassett

**Affiliations:** a College of Natural Science and Mathematics, University of Alaska Fairbanks, Fairbanks, Alaska, USA; b Michael Smith Laboratories and Djavad Mowafaghian Centre for Brain Health, University of British Columbia, Vancouver, British Columbia, Canada; c College of Fisheries and Ocean Science, University of Alaska Fairbanks, Fairbanks, Alaska, USA; d Department of Arctic and Marine Biology, Universitetet i Tromsø–Norges arktiske universitet, Tromsø, Norway; Louisiana State University

## Abstract

The Mesomycetozoea branch near the animal-fungal divergence and are believed to be important to understanding the origins of multicellularity. In 2012, a free-living saprotrophic mesomycetozoean was isolated from the sub-Arctic Bering Sea. A hybrid assembly using Illumina and Nanopore sequences yielded 2,688 contigs with a total length of 125,635,304 bases.

## Abstract (precis)

The Mesomycetozoea branch near the animal-fungal divergence and are believed to be important to understanding the origins of multicellularity. In 2012, a free-living saprotrophic mesomycetozoean was isolated from the sub-Arctic Bering Sea.

## ANNOUNCEMENT

The branching of the Mesomycetozoea near the animal-fungal divergence makes them significant organisms for unraveling the origins of multicellularity (1, 2). Today, only a few Mesomycetozoea genomes have been deposited in public repositories, all of which are animal symbionts. In 2012, a free-living saprotrophic mesomycetozoean strain, Sphaeroforma sirkka B5, was isolated from pollen grains in a nearshore estuarine environment of the sub-Arctic Bering Sea.

We performed a hybrid assembly using long-read Oxford Nanopore minION reads for scaffolding and Illumina HiSeq sequencing for error correction. DNA was extracted using the Mo Bio PowerFood (PF) kit from 48-h-old cells grown on PmTG agar medium with penicillin G and streptomycin (3). DNA was extracted by bead beating twice for 10 min. Lysates were combined on spin filters and subsequently washed twice with PF4 solution. The resulting DNA fragments ranged from 2 kbp to 30 kbp. We purified 5 µg of extracted DNA with AMPure magnetic beads with a bead-to-DNA (vol/vol) ratio of 0.4. We immediately prepared a DNA library with the Oxford Nanopore technology 1D ligation kit (SQK-LSK108) using an optimized protocol. Specifically, an input of 3.7 µg of DNA was used (the shearing step was omitted), and all incubations at 20°C were performed at room temperature. Magnetic bead clean-ups were performed with freshly prepared 80% ethanol solution, and the DNS control strain (DCS) was replaced by 5 µl of nuclease-free water. Sequencing was performed on Oxford Nanopore Mk1 minION system using a R9.4 SpotON FLO-MIN106 flow cell and the 48h_1D_LSK108 script. The device was controlled by minKNOW_1.5.12 implemented on a MacOS system. The library generated 2.19 × 10^6^ reads, with an average size of 6 kbp. The Illumina HiSeq sequencing library was prepared using the TruSeq library kit and loaded on one lane of an Illumina HiSeq 2500 rapid run flow cell (v2). Sequencing was performed with HiSeq Rapid SBS reagents (v2) in a 2 × 100-bp paired-end format.

Nanopore reads were basecalled with Albacore 1.2.4, providing 13.04 Gbp of data subsequently filtered with NanoFilt (4), with a minimum Q score of 10. The final data set (1,141,813 reads with an *N*_50_ value of 7,793 bp, for a total of 7,200,406,494 bp) was assembled using the high-noise robust single-molecule sequencing assembler Canu version 1.7 (5), assuming a genome size of 100 Mbp. Raw contigs were corrected with two iterations of the standalone consensus module Racon. For polishing, two rounds of Pilon (6) were performed with the adapter-trimmed (bbduk and bbtols package) HiSeq data set. Homology search with BLAST on the resulting contigs revealed no contamination. The assembled genome of Sphaeroforma sirkka consists of 2,688 contigs (all greater than 500 bp) with *N*_50_/*N*_90_ values of 76,469/21,989 bp and a total length of 125,635,304 bp. The average contig coverage of the final assembled genome is 180×. Genome completeness was evaluated with BUSCO_V3 (7) using the eukaryote gene set odb9 and Saccharomyces (assessment represented in BUSCO notation), C:84.2% [S:82.5.3%, D:1.7%], F:5.3%, M:10.5%, n:303. The mitochondrion was assembled into a single contig, for a total length of 194,049 bp.

### Data availability.

This whole-genome shotgun project has been deposited in GenBank under the accession number LUCW00000000. The version described in this paper is the third version, LUCW03000000.

## References

[1] Ruiz-Trillo I, Burger G, Holland PWH, King N, Lang BF, Roger AJ, Gray MV 2007 The origins of multicellularity: a multi-taxon genome initiative. Trends Genet 23:113–118. doi:10.1016/j.tig.2007.01.005. 17275133

[2] Ruiz-Trillo I, Lane CE, Archibald JM, Roger AJ 2006 Insights into the evolutionary origin and genome architecture of the unicellular opisthokonts *Capsaspora owczarzaki* and *Sphaeroforma arctica*. J Eukaryot Microbiol 53:379–384. doi:10.1111/j.1550-7408.2006.00118.x. 16968456

[3] Hassett BT, López JA, Gradinger R 2015 Two new species of marine saprotrophic sphaeroformids in the Mesomycetozoea isolated from the sub-Arctic Bering Sea. Protist 166:310–322. doi:10.1016/j.protis.2015.04.004. 26046621

[4] De Coster W, D’Hert S, Schultz DT, Cruts M, Van Broeckhoven C, 2018 NanoPack: visualizing and processing long read sequencing data. Bioinformatics 34:2666–2669. doi:10.1093/bioinformatics/bty149. 29547981 PMC6061794

[5] Koren S, Walenz BP, Berlin K, Miller JR, Bergman NH, Phillippy AM 2017 Canu: scalable and accurate long-read assembly via adaptive *k*-mer weighting and repeat separation. Genome Res 27:722–736. doi:10.1101/gr.215087.116. 28298431 PMC5411767

[6] Walker BJ, Abeel T, Shea T, Priest M, Abouelliel A, Sakthikumar S, Cuomo CA, Zeng Q, Wortman J, Young SK, Earl AM 2014 Pilon: an integrated tool for comprehensive microbial variant detection and genome assembly improvement. PLoS One 9:e112963. doi:10.1371/journal.pone.0112963. 25409509 PMC4237348

[7] Simão FA, Waterhouse RM, Ioannidis P, Kriventseva EV, Zdobnov EM 2015 BUSCO: assessing genome assembly and annotation completeness with single-copy orthologs. Bioinformatics 31:3210–3212. doi:10.1093/bioinformatics/btv351. 26059717

